# UNaProd: A Universal Natural Product Database for *Materia Medica* of Iranian Traditional Medicine

**DOI:** 10.1155/2020/3690781

**Published:** 2020-05-13

**Authors:** Ayeh Naghizadeh, Donya Hamzeheian, Shaghayegh Akbari, Fahimeh Mohammadi, Tohid Otoufat, Saeme Asgari, Azadeh Zarei, Samane Noroozi, Najmeh Nasiri, Mahdi Salamat, Reza Karbalaei, Mehdi Mirzaie, Hossein Rezaeizadeh, Mehrdad Karimi, Mohieddin Jafari

**Affiliations:** ^1^Department of Traditional Medicine, School of Persian Medicine, Tehran University of Medical Sciences, Tehran, Iran; ^2^Department of Biology, Temple University, Philadelphia, PA, USA; ^3^Department of Applied Mathematics, Faculty of Mathematical Sciences, Tarbiat Modares University, Tehran, Iran

## Abstract

**Background:**

Iranian traditional medicine (ITM) is a holistic medical system that uses a wide range of medicinal substances to treat disease. Reorganization and standardization of the data on ITM concepts is a necessity for optimal use of this rich source. In an initial step towards this goal, we created a database of ITM *materia medica*. *Main Body*. Primarily based on Makhzan al-Advieh, which is the most recent encyclopedia of *materia medica* in ITM with the largest number of monographs, a database of natural medicinal substances was created using both text mining methods and manual editing. UNaProd, a Universal Natural Product database for *materia medica* of ITM, is currently host to 2696 monographs, from herbal to animal to mineral compounds in 16 diverse attributes such as origin and scientific name. Currently, systems biology, and more precisely systems medicine and pharmacology, can be an aid in providing rationalizations for many traditional medicines and elucidating a great deal of knowledge they can offer to guide future research in medicine.

**Conclusions:**

A database of *materia medica* is a stepping stone in creating a systems pharmacology platform of ITM that encompasses the relationships between the drugs, their targets, and diseases. UNaProd is hyperlinked to IrGO and CMAUP databases for *Mizaj* and molecular features, respectively, and it is freely available at http://jafarilab.com/unaprod/.

## 1. Background

### 1.1. Iranian Traditional Medicine

Complementary and alternative medicine (CAM) refers to practices of traditional or modern systems that are not considered part of mainstream medicine [1]. Traditional systems of healing, including Chinese Traditional Medicine (TCM), Ayurveda, Unani medicine, and Iranian Traditional Medicine (ITM) or so-called Persian Medicine, have a theoretical foundation and embrace the indigenous knowledge, skills, and practices of ancient civilizations to maintain health, prevent disease, and treat physical or mental illness. The need for an integrative approach to use both complementary and conventional healthcare is increasingly recognized by many countries and allows safe and effective access to traditional medicines [2]. A medical system notable in terms of theory and clinical experience, ITM, is founded on philosophical concepts associated with the four elements [3] (e.g., Table 1). Practiced for thousands of years, ITM is distinctive for world-renowned polymaths such as Rhazes [4] whose book *al-Hawi* (*Continiens*) is an encyclopedia of clinical medicine, embracing the views of predecessor physicians from various countries, and Avicenna whose book, *Canon of Medicine*, was a main medical reference in Europe until the 16^th^ century [5]. Proficient Iranian physicians continued to revise and complement the body of collected knowledge up to the modern period. An exemplar of the 18^th^ century, Mohammad Hosein Aghili, is recognized for books on all scopes of ITM including *Kholasat al-Hekmah* (on principles of Iranian medicine), *Mo*'*alejaat* (on diseases and their treatment), *Makhzan al-Advieh* (on monotherapy), and *Ghrabadin-e Kabir* (on combination therapy).

Though forsaken for nearly a century due to adoption of allopathic medicine, the academic society has recommenced research in CAM recently and integrated it into the healthcare system [6]. ITM takes a personalized approach to health maintenance and healing, considering environmental and patient factors as well as signs and symptoms for disease diagnosis and treatment. Enjoying a holistic view, ITM observes the human body as an integrated system and emphasizes the interconnections between the organs. ITM physicians would consider not just the disease, but more notably the patient to maintain or restore the balance in the body by a number of treatment modalities including lifestyle modification, drugs, and manipulations (massage, bloodletting, etc.).

### 1.2. Modern Medicine

The holistic view in medicine offered by traditional medical schools gradually diminished in the late twentieth century as a result of the emergence of molecular biology, which focuses on the molecular basis of biological mechanisms. Despite all the advances and elucidation of a vast amount of molecular mechanisms, one-disease one-target paradigm has its limits and new trends of pharmacology such as polypharmacology and drug repurposing have developed to cure complex diseases which can rarely be due to an impairment or dysfunction in just one gene or molecule [7]. Recently, the deficiencies of the dominant reductionism in medical research have become more evident, resulting in attention towards systems biology to understand the complex interconnections within biological systems [8–19].

While a system can be defined as a unified whole comprised of regularly interacting or interdependent components [14], systems biology is an attempt to illuminate the whole framework of biological processes by considering all components in the biological entities as a system rather than individual. It facilitates a deeper understanding of the physiologic and pathologic processes as well as recognizing the right targets to treat diseases [17, 20–22].

### 1.3. Integration of Traditional and Modern Medicines

Development of systems medicine and omics technologies has been an aid in providing rationalizations for traditional medicines, proving their efficacy and illuminating the great deal of knowledge they can offer to guide future research in medicine [23–25].

On the other hand, there are several methods to discover new pharmacologically active plant compounds including biodiversity-based [26], chemosystematics [27], ecological [28], computational [29], and ethnopharmacological [30] approaches. Exploiting ethnomedical data, which includes both folklore and organized traditional medical systems, is a time and cost-effective method to discover new pharmacological compounds with higher hit-rate compared with biodiversity-based methods [31, 32]. In fact, databases of traditional medicine can be a valuable resource for new drug discovery. For example, TCM scientists have addressed this issue and developed a number of databases such as traditional Chinese medicine integrated database (TCMID) [33, 34] or collective molecular activities of useful plants (CMAUP) [35], which are host to data on all aspects of TCM, including diseases, drugs, and their targets. These databases also provide external links to other resources including OMIM (Online Mendelian Inheritance in Man), Drugbank, PubChem, and STITCH (search tool for interactions of chemicals) [33].

However, there was lack of ITM databases available to the academic society. An apt starting point would be creating a database of *materia medica,* one of the key features in traditional medical systems including ITM. The body of collected knowledge on the therapeutic characteristics of herbal, animal, and mineral compounds has been extensively documented and revised by ITM physicians throughout the millennia [36]. Properties described for medicinal substances are determined through inductive reasoning from foundational principles of ITM (for example, deeming certain drug actions for monographs with a hot Mizaj), as well as the clinical experience of physicians. In order to exploit the full potential of traditional medical resources, their data and concepts should be reorganized and standardized to create a shared interface with conventional medicine.

To address the data integration problem, we created Universal Natural Products Resource (UNaProd), a database of medicinal substances consisting of 2696 drug monographs based on an encyclopedia of ITM *materia medica*, i.e., *Makhzan al-Advieh*, a foundation on which more comprehensive databases of ITM can be built. This database will help exploit knowledge from ITM resources in a number of ways. Firstly, ITM may be expressed in a terminology that can be used by modern medicine. Second, the relationships between ITM concepts will be more precisely determined, which will in turn unveil the underlying biological system of this medical school. Third, providing justifications for traditional use of plants by linking them to their associated compounds will facilitate integration of ITM with both allopathic medicine and other traditional medical systems including TCM. Fourth, it will provide an extensive range of options for discovery of new drugs. As a time- and money-consuming process, new drug discovery will indeed be facilitated through utilizing the connections ITM has established between a medicinal substance and a specific disease.

### 1.4. Construction and Content

**Figure 1:**
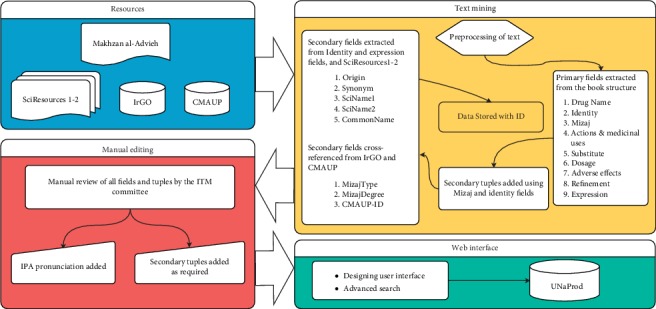
UNaProd construction pipeline. The database was created by text mining Makhzan al-Advieh to extract the primary fields from the text and secondary fields from SciResources, IrGO, and CMAUP. Subsequently, all fields were manually reviewed, and secondary tuples were added to the database. In the next step, the web interface was designed to create an online database.

UNaProd database was created by means of both text mining and manual editing by an ITM committee comprised of specialists and researchers in the Department of Persian medicine, Tehran University of Medical Sciences. The construction pipeline is illustrated in Figure 1.

## 2. Resources

### 2.1. Makhzan Al-Advieh

Among the resources available in ITM concerning documentations of medicinal substances, *Makhzan al-Advieh* written by Mohammad Hosein Aghili in 1769 A.D. is the most recent encyclopedia of *materia medica* and contains the greatest number of drug monographs; thus, it was selected as the resource for the raw data of our database. The *Makhzan al-Advieh* manuscript consists of an introduction describing the contents of the book and thirteen chapters and an appendix providing synonyms and translations. Twelve chapters are on preliminary topics including term definitions, estimation of drug *Mizaj*, units of weight, instructions on drug preparations, and astronomical considerations. The main chapter of the book is comprised of drug monograph documentation in alphabetical order. Drug monographs are described in terms of synonyms, identity, *Mizaj*, actions and medicinal uses, dosage, adverse effects, refinement, and substitute in this semistructured book. Furthermore, Aghili has mentioned viewpoints of other physicians and pharmacologists in the cases where there is no consensus on a certain characteristic or when prominent scholars like *Avicenna*, *Galen*, and *Ibn-e Baitar* have other opinions.

The initial part of each drug monograph description includes a description of its correct pronunciation, synonyms, and translations. About 70 languages and dialects have been used to provide translations, the most common of which include Persian, Arabic, Indian, and Greek. A description of the identity of each drug monograph in terms of its origin, types, and morphological characteristics is provided in the second part. Most drug monographs are of either herbal, animal, or mineral origin, but there are also instances of compound drug monograph like *Maligharaten*, which is honey boiled in water. The types and varieties of the medicinal substance, for example, being wild or cultivated for plants and wild or domestic for animals, the morphological characteristics of drug parts such as leaves, bark, flower, or fruit, and the place of origin including countries and geographical locations, where the drug monograph may be found, are included in this section. In the cases where the identity of a drug is in dispute, Aghili has mentioned views of different scholars. The third part of each drug monograph is dedicated to *Mizaj*, which is a key concept in Iranian traditional medicine and an attribute of all that exists in the universe. Persian scholars determined the *Mizaj* of a medicinal substance through deduction and experience [37]. Subsequently, actions and medicinal uses, which encompass a list of pharmacological actions followed by details on the medical conditions which benefit from the drug, are discussed. Adverse drug reactions, refinements, dosage, and substitute comprise the succeeding parts of drug monograph documentation. Adverse drug effects include the body organs or individuals that may be harmed by the drug and is usually followed by refinement, which includes particular drug preparation or another drug to reduce or eliminate the mentioned side effects. Dosage has been provided for some of the drugs, and for more potent ones, the lethal dose has also been specified. A substitute, which is a drug with similar effects that can be used as a replacement, is included for some monographs. The potency of the substitute drug has been reported as the relative proportion that should be prescribed to reach a similar effect.

### 2.2. Resources for Scientific Names

Two resources were used to extract scientific names of the herbal substances. Composed by *Ghahreman* and *Okhovvat*, the first resource (“*SciResource1*”) titled “Matching the Old Medicinal Plant Names with Scientific Terminology,” includes a history of medicinal plants, the methodology of using ancient texts on medicinal plants, the old medicinal plant names, and the identification of scientific names for plants [38]. The second resource (“SciResource2”) includes the appendix provided in the corrected edition of Makhzan al-Advieh (“MakhzanCE”), from which both scientific names and common names were extracted [37].

### 2.3. Iranian Traditional Medicine General Ontology (IrGO)

IrGo [39], which currently consists of ontology terms for *Mizaj*, was used as the resource for the *Mizaj* type and degree provided in *Makhzan al-Advieh*.

### 2.4. Collective Molecular Activities of Useful Plants Database (CMAUP)

Additionally, UnaProd was linked to plants with scientific names existing in CMAUP, a database covering a total of 5645 plants in medicinal, food, edible, agricultural, and garden plant classes. CMAUP also provides drug targets (ChEMBL target classes) and their activity levels, together with GO categories, KEGG categories, and ICD blocks for gene ontologies, biological pathways, and international classification of diseases, respectively.

## 3. Field and Tuple Extraction

### 3.1. First Phase: Text Mining

Text mining methods were used to generate the primary fields in UNaProd database. Initially, the text of *Makhzan al-Advieh* was preprocessed. There are a number of Arabic characters, including *Tanwin* (اً) and *Hamzah* (ئ) that are used in different formats in Persian texts; thus, these Arabic diacritics were removed and replaced by simple *Alef* (ا) or *ye* (ی) of the Persian alphabet to facilitate searching. Also, there are two unicodes for the letters *kaf* (ک) and *ye* (ی), which can be used interchangeably and thus needed to be normalized. To perform the abovementioned tasks, Hazm package in Python was used [40]. Additionally, some prefixes and suffixes including “می” (ing) in verbs, can be written without a separator (“میگوید”), or be separated from the verb either by a space (“می گوید”) or zero-width space (“میگوید”). Since zero-width space is not routinely used by all of the books and is not possible to type in browsers, all such cases were unified and changed to a space.

Each drug and its description were separated using regular expressions and stored in the “Drug” table. Aghili has used certain words to demarcate the beginning of each part in the drug monograph description, which were used to separate the information of each field. Following extraction of the mentioned fields from the text, the string (a sequence of characters) not pertaining to any of those keywords was labelled as the “Expression” field of that drug, which mainly consisted of the pronunciation and synonyms. The descriptive pronunciations in the “Expression” field were manually translated into International phonetic alphabets (IPA) and stored in the “Pronunciation” field. The abovementioned fields, namely, “DrugName”; “Mizaj”; “Identity”; “Substitute”; “AdverseEffects”; “Actions&MedicinalUses”; “Dosage”; and “Remarks” constituted the primary fields for each drug monograph. To determine the synonyms provided for each drug monograph, a list comprising all languages, dialects, and scholar names mentioned in the text was prepared, and subsequently, terms that followed the words in this list in the “Expression” and “Identity” fields were determined as the synonyms of drug monographs and stored in the “Synonym” table.

Secondary fields for each tuple include ‘Pronunciation', “Synonym,” “Origin,” “CommonName,” “SciName1,” “SciName2,” “MizajType,” and “MizajDegree.” The “Origin” field of a drug, whether “animal,” “herbal,” “mineral,” “compound,” or “other,” was determined based on the existence of related keywords in the “Identity” field. In most cases, the authors explicitly mentioned the origin in the first sentence of this field. Otherwise, the keywords of each origin type were matched in the remaining parts of the identity of a drug. Eventually, an origin type for each drug was assigned based on the frequency of the keywords for each group, namely herbs, animals, minerals, or compounds. In other words, the origin type with the most keywords in the ‘Identity' field was assigned to the drug. Fields of “CommonName,” “SciName1,” and “SciName2” for plants were determined by text mining “MakhzanCE,” “SciResource1,” and “SciResource2,” respectively. Animal and mineral scientific and common names were manually curated. Plants with scientific names were linked to the CMAUP database.

The *Mizaj* of each drug was extracted using IrGO (Iranian traditional medicine General Ontology), and stored in a separate table called “DrugMizaj.” Currently consisting of an ontology for *Mizaj*, IrGO was used to determine the *Mizaj* type and degree of drug monographs based on the existence of the keywords in the “Mizaj” field. For each drug with a “Mizaj” description, one or more rows, depending on whether other scholars' views were quoted in the text, were added to “DrugMizaj.” The resulting table contains information regarding the ID of drugs, the scholar from whom the specified *Mizaj* was quoted, “*MizajType*” and hotness, coldness, wetness, and dryness degrees collectively named “*MizajDegree*.” The extracted types and degrees were linked to the IrGO database.

### 3.2. Second Phase: Manual Editing

Following extraction of the abovementioned fields, all parts of the resultant database was reviewed manually by the ITM committee and verified with the lithograph print of Makhzan al-Advieh. Regarding many drug monographs, Aghili mentions distinct characteristics for drug parts and different types, varieties, or processed form of drugs. One of the examples is quince, in which distinct *Mizaj* actions and medicinal uses have been attributed to its blossom, seed, sweet fruit, sour fruit, and purée prepared from fruit; also, features of a processed form, quince oil, have been specified. Thus, in order to create a more organized and easier access to the database, some secondary tuples were extracted from the primary ones.

## 4. Web Interface

UNaProd was built using PHP 7.2.11 for server-side data processing, Javascript ECMAScript 2015, for the frontend and Plotly library 1.40.0 for the generation of the interactive visualizations while data were stored in MySQL 10.1.37. An introduction to the database, together with a manual and statistics, is available in the website. Basic and advanced search options were also provided to enable query of database contents by one or more keywords in the fields selected by the users from dropdown menus.

## 5. Utility and Discussion

### 5.1. Database Structure

Based on the primary fields, UNaProd database contained 1741 drug monographs which increased to 2696 by manually creating the secondary tuples. In addition to “Identity,” “*Mizaj*,” “ActionsandMedicinalUses,” “Dosage,” “AdverseEffects,” “Refinements,” “Substitute,” and “Expression,” which constitute the primary fields of the database extracted from the text of *Makhzan al-Advieh*, three fields including two columns of scientific names, a column of common name, were extracted from resources other than the text. An origin was ascribed to each drug based on the identity text fields (Figure 1). *Mizaj* types and degrees of drug monographs were extracted using IrGo database and stored in a separate “DrugMizaj” table. Furthermore, synonyms mentioned by other scholars or translations were extracted from the “Expression” and “Identity” fields and stored in a separate “Synonyms” table. The diagram of the resultant database was depicted in Supplementary file 1.

### 5.2. Drug Origins

**Figure 2:**
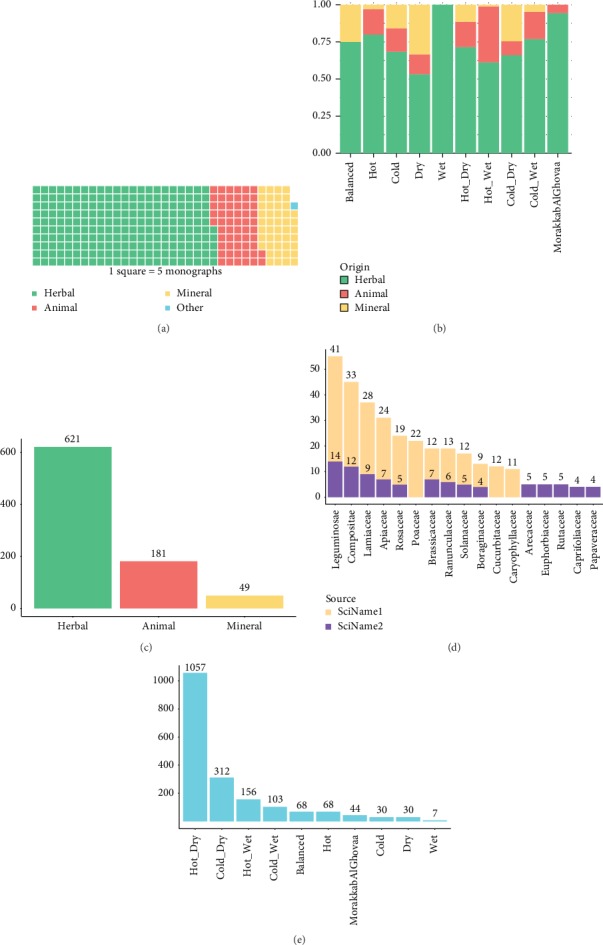
(a) Frequency of each origin (herbal, animal, mineral, and others) in UNaProd database; (b) the share of each origin in the main ITM Mizaj types. (c) Frequency of scientific names extracted for drugs with herbal, animal, and mineral origins. (d) The most common plant families of UNaProd extracted from each of the scientific name resources. (e) Frequency of the main Mizaj types of drug monographs in UNaProd. The numbers on each bar represent the exact number of frequencies.

Drugs were defined to have herbal, animal, mineral, or other origins, the percentages of which and their share in the main *Mizaj* types were shown in Figures 2(a) and 2(b). Herbs clearly constituted the majority of drug monographs, followed by medicinal substances with animal and mineral origin that comprise approximately 17% and 14% of the database, respectively. Approximately 0.4% (6 drugs) had origins other than those mentioned above, which consisted of those that encompass drugs of any origin or drug monographs with no consensus on the origin. For example, “*So*'*out*” is a general term including any drug that is insufflated through the nose. Another example is “*Ghavand*,” a drug of ambiguous origin. As Aghili has stated, some scholars have described it as an herbal oil, while others have believed it to be an oil of animal origin. Collectively, herbs constituted the most instances in all of types of *Mizaj*, whilst medicinal substances with an animal origin were mostly hot-wet, and minerals typically had a dry, cold-dry, or balanced *Mizaj*.

### 5.3. Scientific Names

Scientific names from the SciResources 1-2 were stored in two separate fields. Collectively, a total of 1822 drug monographs possess scientific names from one or both resources.  Figure 2(c) provides information on the number of scientific names. As demonstrated, a total of 851 monographs including 621 herbs, 181 drugs with animal origin, and 49 minerals had scientific names. There are 66 and 114 different families based on “SciName1” and “SciName2,” respectively.  Figure 2(d) shows the most common plant families extracted from the two resources with the highest frequency seen in *Leguminosae*, *Compositae*, *Lamiaceae*, and *Apiaceae* families, respectively. *Leguminosae*, commonly known as *Fabaceae* or pea family, is widely distributed with approximately 730 genera and 19,400 species [41]. This richness of this species is reflected in great morphological and chemical diversity, from which numerous compounds are derived [42]. The outstanding feature of this family is the presence of roots tubercles or nodules which harbor nitrogen-fixing bacteria (*Rhizobium*). Pharmacological actions of these plants include analgesic, anti-inflammatory, antiulcer, anticancer, antidiabetic, anti-inflammatory, antirheumatic, antimicrobial, antibacterial, and cytotoxic activities [43, 44]. The Compositae (*Asteraceae*) is one of the largest and most diverse families of flowering plants. They are found in diverse habitats and contains over 40 economically important species including food (lettuce and Jerusalem artichoke), oil (sunflower and safflower), medicinal (chamomile), and ornamental (*Chrysanthemum*, *dahlia*, *zinnia*, and *marigold*) crops. Many members of *Asteraceae* with pharmacological activity contain important phytochemical compounds such as polyphenols, flavonoids, and diterpenoids and have antibacterial, antifungal, anti-inflammatory, insecticide, and antitumor capacities of *Asteraceae* species [45, 46]. *Lamiaceae*, also known as the mint family, is a large plant family of mostly shrubs and herbs containing about 236 genera and 6,900 to 7,200 species. *Lamiaceae* are distributed globally with particularly high diversity in the Mediterranean region. Medicinal properties of the *Lamiaceae* species are often attributed to their high content of volatile compounds. Many members of the family including Ajuga, Coleus, and Salvia are widely cultivated as ornamentals, while others such as sage (*Salvia*), thyme (*Thymus*), mint (*Mentha*), marjoram (*Origanum*), rosemary (*Rosmarinus*), lavender (*Lavandula*), and basil (*Ocimum*) are widely used as culinary herbs and spices [47]. The *Apiaceae* or carrot family consists of plants with a characteristic umbrella-arranged fruit. Comprising of approximately 450 genera and 3700 species, this family of plants can be found worldwide [48]. The noteworthy character of this family is the high content of volatile oil or oleoresin [49]. Members of *Apiaceae* possess various compounds with many biological activities. Some of the main properties are antibacterial, hepatoprotective, vasorelaxant, and antitumor activities as well as the ability to induce apoptosis and inhibit cyclooxygenase [50, 51]. Other medicinal herbs with high commercial value can be especially found in *Solanaceae*, *Boraginaceae*, *Cucurbitaceae*, *Euphorbiaceae*, and *Papaveraceae* plant families.

### 5.4. Drug *Mizaj*

The *Mizaj* type and degree for every drug monograph with a *Mizaj* description in *Makhzan al-Advieh* were determined based on IrGO (Iranian traditional medicine General Ontology) and thereafter reviewed manually by the ITM committee. Users can view the attributes of the specified *Mizaj* type and degree provided for each drug, as these fields were linked to the IrGO database. Moreover, in the instances where Aghili has quoted a different *Mizaj* from other physicians and pharmacologists, the name of the scholar together with the *Mizaj* type they have specified was provided.

Aghili has described a *Mizaj* for 1421 of the 1741 main drug monographs, including 384 views from other scholars. Considering the 2696 tuples of UNaProd, a total of 1976 tuples contain information regarding *Mizaj*. There are ten main *Mizaj* types in ITM, the frequency which was demonstrated in Figure 2(e). The most common *Mizaj* type for medicinal substances of *Makhzan al-Advieh* was the hot-dry *Mizaj* constituting 55.7% of the drug monographs with a determined *Mizaj*, followed by cold-dry (16.5%), hot-wet (8.1%), cold-wet (5.4%), balanced (4.8%), hot (3.6%), and *Morakkab al-Ghovaa Mizaj* ascribed to drugs that can exhibit more than two qualities (2.3%), dry (1.7%), cold (1.6%), and finally wet (0.4%) *Mizaj* types.

**Figure 3:**
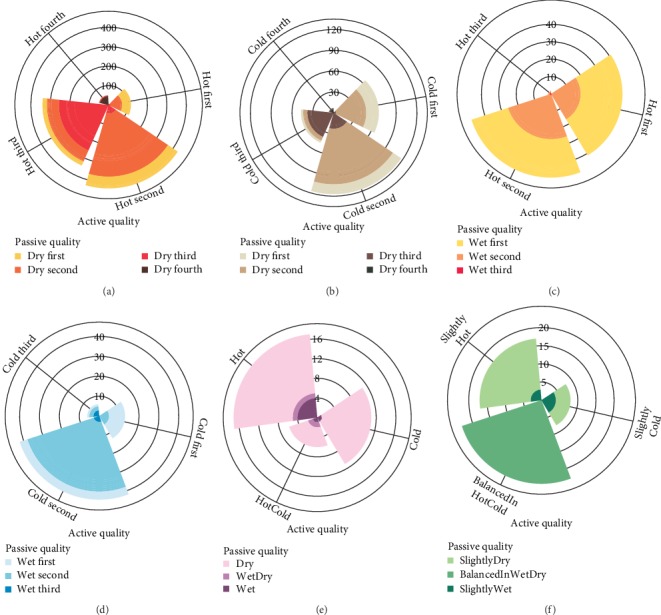
Mizaj degrees based on active (hotness/coldness) versus passive (wetness/dryness) qualities in Mizaj types of UNaProd drug monographs. In all plots, the *x* axis is the active degree and the *y* axis is the count of drug monographs. (a) Hotness degree versus dryness degree in drugs with hot-dry Mizaj; (b) coldness degree versus dryness degree in drugs with cold-dry Mizaj; (c) hotness degree versus wetness degree in drugs with hot-wet Mizaj; (d) coldness degree versus wetness degree in drugs with cold-wet Mizaj; (e) active degree versus passive degree in drugs with balanced Mizaj; (f) active degree versus passive degree in drugs with Morakkab al-Ghovaa Mizaj.

Regarding *Mizaj* degrees, ITM specifies an active and a passive quality for substances. Active qualities include hotness or coldness, while wetness and dryness constitute passive qualities. The potency of each quality is described in a range from first to fourth. The frequency of drug monographs with *Mizaj* types which have both qualities specified, i.e., hot-wet, hot-dry, cold-wet, and cold-dry; balanced; and *Morakkab al-Ghovaa Mizaj* types, have been illustrated in Figure 3. Hot-dry drugs were mostly second or third degree in hotness (Figure 3(a)), while for cold-dry and cold-wet drugs, both active and passive qualities were in second degree (Figures 3(b) and 3(d)), and the hotness and wetness in hot-wet drugs generally did not exceed second degree (Figure 3(c)). The most common qualities in drugs with a balanced *Mizaj* inclined towards slight hotness and dryness (Figure 3(e)). In drugs with *Morakkab al-Ghovaa Mizaj*, the passive quality of dryness was specified more prominently than wetness (Figure 3(f)).

**Figure 4:**
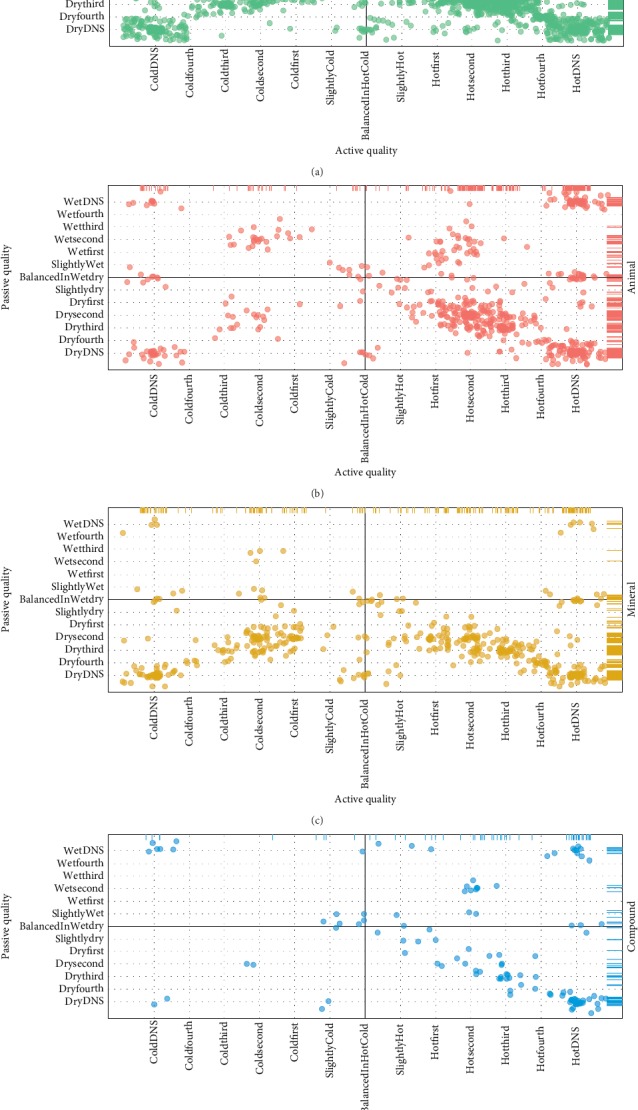
Mizaj jittered scatterplots of UNaProd drug monographs in terms of active versus passive quality faceted by origin. (a) Herbal drug monographs; (b) animal drug monographs; (c) mineral drug monographs; (d) compound drug monographs. The two perpendicular lines in each plot indicate balance in either axis. The active quality consists of the main degrees of hotness and coldness, while passive quality includes the main degrees from fourth degree in dryness to fourth degree in wetness. As some Mizaj descriptions do not contain the exact degree, a further factor-DNS (degree not specified) has been defined for each quality.

The *Mizaj* type and degree of UNaProd drug monographs have been also demonstrated in Figure 4 in more detail. The active quality lies in a range from coldness in maximum fourth degree to hotness in maximum fourth degree, while passive quality ranges from dryness in maximum fourth degree to wetness in maximum fourth degree. There are a number of instances where Aghili has only described the *Mizaj* type but not the degree. For example, *Khatai* tea has a hot and dry *Mizaj* but neither the degree of hotness nor dryness has been specified. To demonstrate these drug monographs in the scatter plot, DNS factors have been added to each axis (Table 1). Out of the total 1249 hotness degrees and 441 coldness degrees, 448 and 227 were unspecified, respectively. Regarding passive qualities, there were 148 and 442 unspecified degrees out of the 386 and 1480 existing degrees for wetness and dryness degrees, respectively.

### 5.5. User Interface

**Figure 5:**
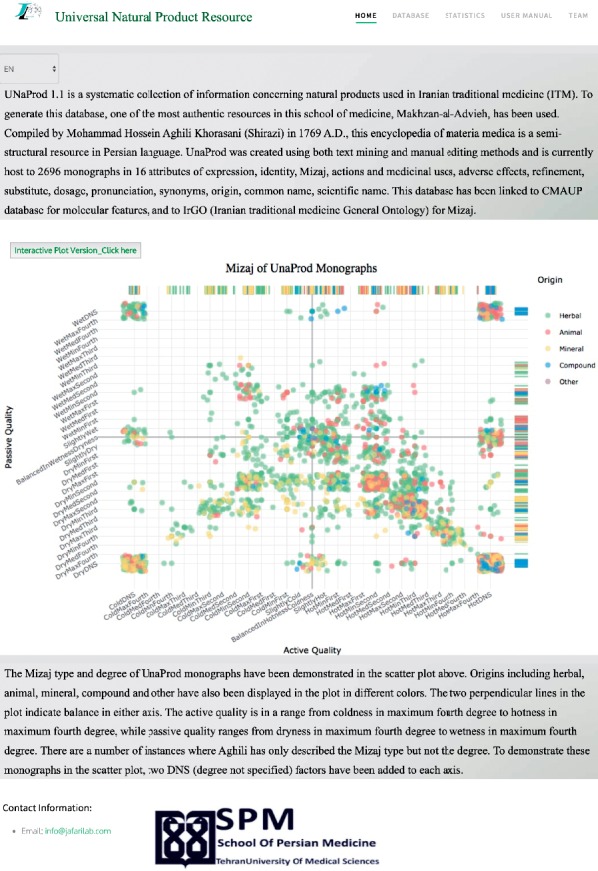
UNaProd home page including an introduction to UNaProd along with the following features: the “database” link; a “user manual”; and “Statistics.”

The home page of UNaProd (Figure 5) database accommodates an introduction to UNaProd as well as the following features: (a) the “database” link enables users to browse the drugs and their specifications via a table containing all fields; a search bar has been incorporated in this page that allows search in a specific field or all of the database; (b) a “user manual” section describes the general structure of the database along with an explanation of each field and the keywords by which they have been extracted from the book; (c) “Statistics”: information regarding the integrity of the database and also statistics on the origin and Mizaj fields and the languages for which synonyms have been mentioned and can be accessed via a separate link in the database. The database is freely available to all users with no restriction at http://jafarilab.com/unaprod/.

## 6. Conclusions

As a traditional medical system with a prolific history, ITM contains knowledge that can be better utilized if promoted using modern systems medicine approaches. Meanwhile, the rationale behind physiologic or pathologic processes can nowadays be made possible by analysis of complex networks [17]. Due to the holistic nature of ITM, deducing justifications for the concepts of the personalized approach to maintain health and treating diseases requires establishing links to modern medicine. Thus, standardization and organization of ITM concepts and drugs shall undoubtedly facilitate the validation of its theories, as has been made possible with other traditional medical systems [33].

Systematization of ITM and construction of an open knowledgebase, which can subsequently be linked with modern biological databases, can help fulfill the increasing demands of researchers in achieving a deeper understanding of the concepts and mechanisms of ITM. We have endeavored to take a step towards fulfilling this goal by creating a database of *materia medica*, UNaProd.

The knowledge and experience provided by traditional systems of medicine can help reduce the expenses, time consumed, and also toxicity occurrences in developing new drugs from natural sources [31, 52]. Thus, considering all the challenges and financial issues in the pharmaceutical industry, an effective utilization of this database may facilitate the drug discovery process from ethnomedical data. This process starts with selecting a drug from the *materia medica* [53], which may be accelerated by a database considering the large number of available information based on ITM.

Since IrGO ontologies include English translations and definitions for ITM concepts, we intend to link our database to ontologies of IrGO as they develop to make the database usable for non-Persian speakers. This will also be of aid in creating a systems pharmacology platform of ITM *materia medica* that encompasses the relationships between the drugs, their targets, and diseases and constructing a network of the information provided in the ITM literature. We will endeavor to expand the database and include other resources of ITM *materia medica* and also provide links to phytochemical and other traditional medicine databases.

## Figures and Tables

**Table 1:** Some basic definitions of ITM.

Term	Definition
Qualities	The four qualities of hotness, coldness, wetness, and dryness are foundational concepts in ITM, by which phenomena including the effects of medicinal substances on the body are explained.
Active quality	Hotness and coldness are considered effectual on wetness and dryness and thus called active qualities.
Passive quality	Wetness and dryness are considered affectable by hotness and coldness and thus called passive qualities.
*Mizaj*	*Mizaj* is defined as the uniform quality, created via interaction of the four qualities constituting a substance in various proportions. A prediction of the effects of medicinal substances on the body is possible via this property.
*Mizaj* type	Described as either balanced or unbalanced and expressed as exclusively one of the qualities or a combination of an active and a passive quality.
*Mizaj* degree	The potency of each of the qualities in a *Mizaj* type is mostly expressed in a range between one and four (least to most potent), each further classified into minimum, medium, or maximum.
DNS (degree not specified)	In UNaProd database, DNS is ascribed to qualities of *Mizaj* for which a *Mizaj* type has been determined, while no degree has been specified.

## Data Availability

The data used to support this study are publicly available at http://jafarilab.com/unaprod/

## Abbreviations

ITM: Iranian traditional medicine
CAM: Complementary and alternative medicine
TCM: Chinese traditional medicine
IPA: International phonetic alphabets
IrGO: Iranian traditional medicine General Ontology
UNaProd: Universal Natural Product Database.

## Supplementary Materials

Supplementary Materials The diagram of the resultant database is depicted in Supplementary File 1.
