# Supplementary figures and images for: UNaProd: A Universal Natural Product Database for Materia Medica of Iranian Traditional Medicine
Source: Evid Based Complement Alternat Med. 2020 May 13;2020:3690781. doi: 10.1155/2020/3690781 (PMC7243028; doi:10.1155/2020/3690781)

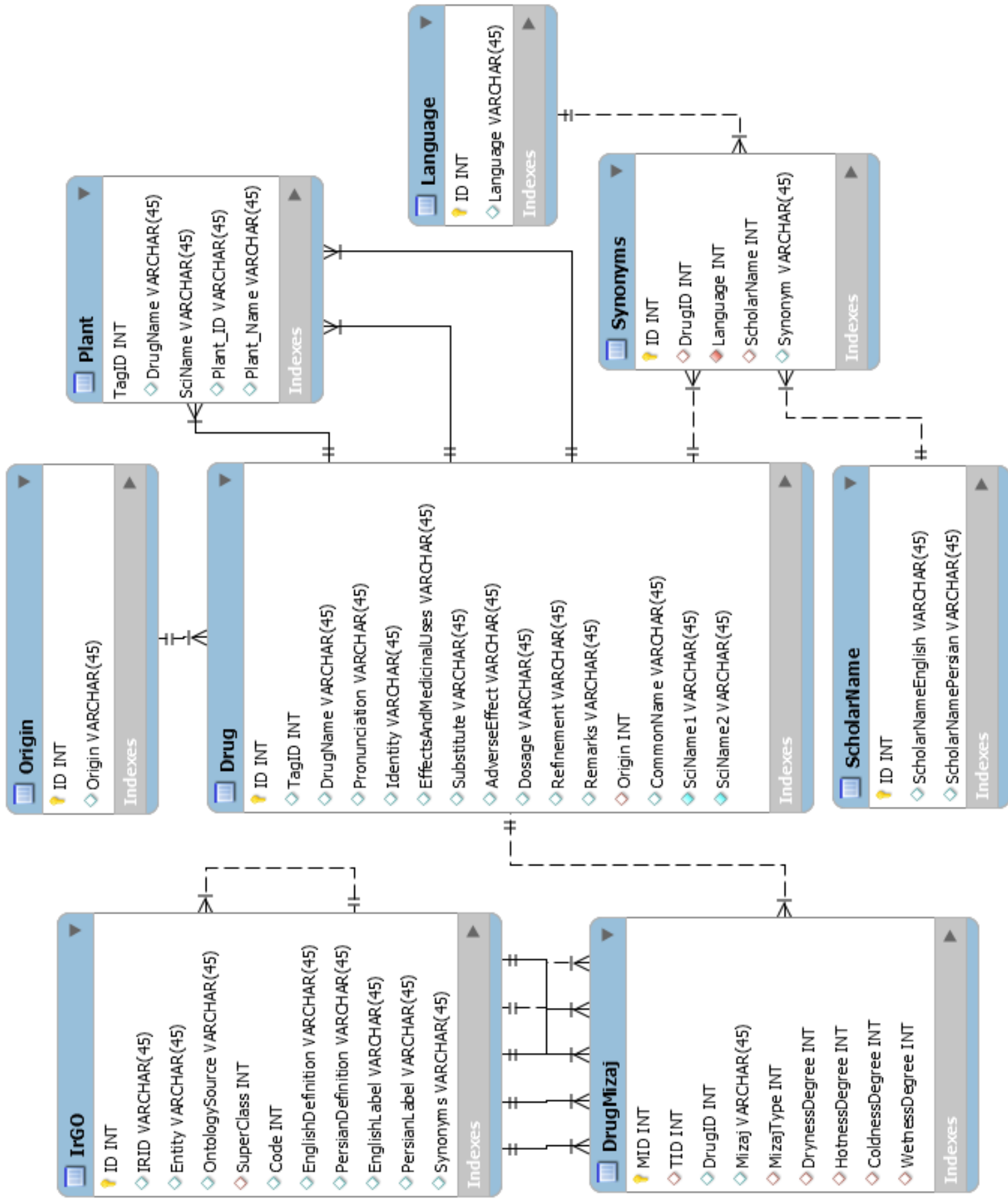

Supplement: Supplementary Materials — The diagram of the resultant database is depicted in Supplementary File 1. [file 3690781.f1.pdf]
